# Short-Term Exposure to Warm Microhabitats Could Explain Amphibian Persistence with *Batrachochytrium dendrobatidis*

**DOI:** 10.1371/journal.pone.0026215

**Published:** 2011-10-18

**Authors:** Joshua H. Daskin, Ross A. Alford, Robert Puschendorf

**Affiliations:** School of Marine and Tropical Biology, James Cook University, Townsville, Queensland, Australia; Smithsonian's National Zoological Park, United States of America

## Abstract

Environmental conditions can alter the outcomes of symbiotic interactions. Many amphibian species have declined due to chytridiomycosis, caused by the pathogenic fungus *Batrachochytrium dendrobatidis* (*Bd*), but many others persist despite high *Bd* infection prevalence. This indicates that *Bd*'s virulence is lower, or it may even be a commensal, in some hosts. In the Australian Wet Tropics, chytridiomycosis extirpated *Litoria nannotis* from high-elevation rain forests in the early 1990 s. Although the species is recolonizing many sites, no population has fully recovered. *Litoria lorica* disappeared from all known sites in the early 1990 s and was thought globally extinct, but a new population was discovered in 2008, in an upland dry forest habitat it shares with *L. nannotis*. All frogs of both species observed during three population censuses were apparently healthy, but most carried *Bd*. Frogs perch on sun-warmed rocks in dry forest streams, possibly keeping *Bd* infections below the lethal threshold attained in cooler rain forests. We tested whether short-term elevated temperatures can hamper *Bd* growth *in vitro* over one generation (four days). Simulating the temperatures available to frogs on strongly and moderately warmed rocks in dry forests, by incubating cultures at 33°C for one hour daily, reduced *Bd* growth below that of *Bd* held at 15°C constantly (representing rain forest habitats). Even small decreases in the exponential growth rate of *Bd* on hosts may contribute to the survival of frogs in dry forests.

## Introduction

The distributions, abundances, life history strategies, and virulences of microbial symbionts can all be influenced by the environment, and can, in turn, affect the development and outcome of disease [1], [2], [3]. *Batrachochytrium dendrobatidis (Bd)*, the organism that causes chytridiomycosis, has been linked to the decline and potential extinctions of hundreds of species of amphibians around the globe [4], [5]. In the tropics, higher elevation rainforest sites, which correspond to areas of high amphibian diversity and endemism, have been especially hard hit by this disease [6], [7], [8]. The cooler temperatures in these habitats coincide with the *in vitro* thermal optimum for growth of this pathogen [9], [10]. In the laboratory, frogs infected with *Bd* can consistently lose their infections after relatively short (16 h) exposures to high temperatures (37°C), as the fungus perishes rapidly at this temperature [11]. Constant temperatures as low as 28–30°C resulted in death of the fungus after several days in culture [9], suggesting that even small elevations in environmental temperature are likely to tip the host-pathogen balance in favor of the host. However, the response of *Bd* to realistic thermal regimes experienced by persisting and declining populations in the wild has not been tested.

In the Australian Wet Tropics, the upland-endemic torrent frog *Litoria lorica* experienced severe population declines due to *Bd* at all known localities, and was last seen in 1991 [12], [13], [14]. By the mid-2000s it was thought to be extinct. However, in 2008 this species was rediscovered at a previously unknown locality in upland dry forest, where it occurs at relatively high local abundance, despite high prevalences of *Bd* infection. The newly-discovered site is approximately 6 km downstream from rain forest sites at which the species had been extirpated by chytridiomycosis. Another torrent frog, *Litoria nannotis*, declined from most high elevation rainforest sites, is sympatric with *L. lorica*, and occurs in high abundance with high prevalences of *Bd* infection at the *L. lorica* rediscovery site [15]. These species inhabit waterfalls and torrents, spending their days in and under the water and emerging onto adjacent rocks late in the day at the start of their nocturnal activity periods [16], [17], [18]. Puschendorf et al. [15] hypothesized that the coexistence of *L. lorica* with a potentially lethal pathogen in the dry forest was linked to the lack of canopy. Because infections must reach a threshold intensity before mortality occurs [19], [20], dry forest frogs may reduce infection intensity by effectively “basking” on warm rocks after emerging from their diurnal shelters, killing or greatly reducing the growth rate of the fungus and avoiding mass mortality due to the disease [15]. There is evidence for similar behavioral regulation of infection status in at least one neotropical anuran [21].

To test this hypothesis, we collected substrate temperatures experienced by *L. nannotis* in open dry forest environments and adjacent rainforest. Based on these temperatures we carried out an *in vitro* experiment in which we grew *Bd* under thermal regimes simulating the thermal environments *L. lorica* experience at upland rain forest and dry forest sites. This is the first study to test *Bd*'s response to temperature regimes experienced by diseased and persisting amphibian populations.

## Methods

We used ten temperature dataloggers (DS1921Z-F5, Dallas Semiconductor, Dallas, Texas USA) placed on rocks that *L. lorica* perch on in the dry forest and eight dataloggers at sites that *L. nannotis* perch on in nearby rainforest (details of sites appear in Puschendorf et al. 2011). Temperatures were recorded in synchrony every half hour from 23/8/2010 (22∶00) to 17/09/2010 (8∶30).

In the laboratory, *Bd* was grown in three temperature regimes, based on the rock temperatures recorded in the field. *Bd* (isolate Gibbo River, L. Les donna, 06-LB-1) was flushed from ½-strength TGhL (eight g tryptone, one g gelatin, one g lactose, and 10 g bacteriological agar per liter of water) agar plates using three mL of ½-strength TGhL and filtered to remove sporangia. 3.5×10^4^ zoospores were inoculated into 100 µL ½-strength TGhL in each of 30 wells of each of three 96-well assay plates, a method modified from that of Rollins-Smith et al. [22]. All plates were kept at 15°C for the first 24 hours. Thereafter, one plate was kept at 15°C 24 hours a day to simulate constant, cool conditions at rain forest sites, a second was kept at 15°C 23 hours a day with one hour at 28°C to simulate daily exposure to moderately-warmed rocks as frogs emerge from diurnal retreat sites in dry forests, and a third was kept at 15°C 23 hours a day with one hour at 33°C to simulate emergence onto warmer rocks in dry forests. Treatments are hereafter referred to as 1) rain forest control, 2) dry forest 28°C spike, and 3) dry forest 33°C, respectively.

The growth of *Bd* cultures was measured spectrophotometrically at 492 nm [22] at the outset, and every 24 hours thereafter, immediately after the higher-temperature plates were exposed to their treatments. Cultures were also monitored visually using an inverted light microscope to observe growth and check for contamination.

The initial optical density at 492 nm (OD_492_) for each well was subtracted from each subsequent reading to give the adjusted OD_492_, as change since time zero. To test for differences in *Bd* growth among treatments, a one-way ANOVA with Tukey's post-hoc tests was performed on the final adjusted OD_492_ for each treatment. Analysis was performed in SPlus 8.0 for Windows (Insightful Corporation, 2007).

We used laboratory assays rather than *in vivo* experiments because both species are endangered (*L. lorica* is critically endangered).

## Results

**Figure 1:**
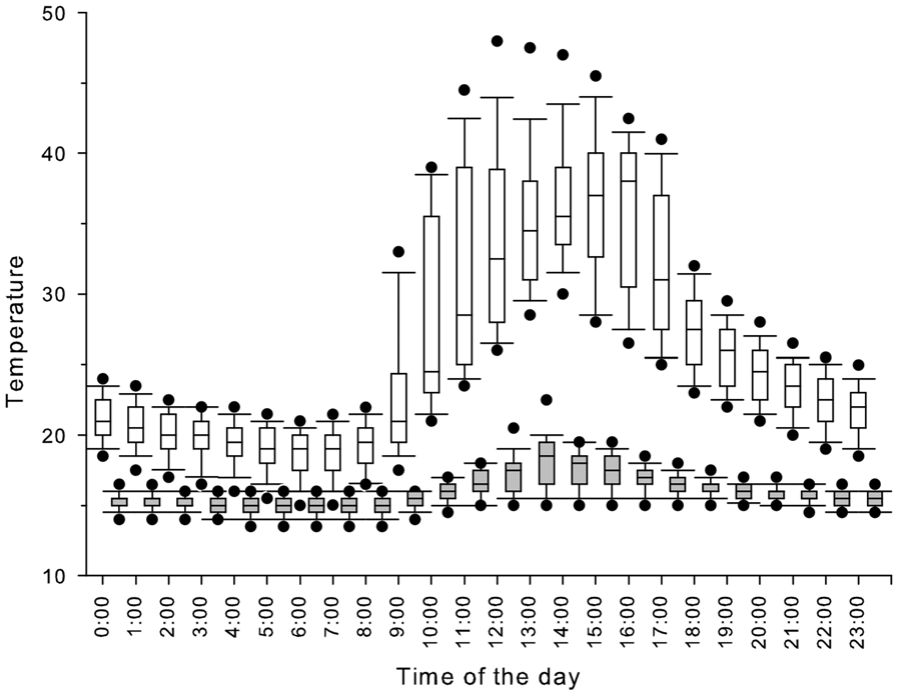
Perch temperatures available for torrent frogs in dry forest (open boxplots) and rainforest (grey boxplots). Boxplots illustrate the distribution of temperatures recorded at each time by all dataloggers in a habitat on all sampling days. Horizontal lines indicate medians, boxes display the interquartile (IQ) range, and whiskers show the range of temperatures within 1.5 times the IQ range. Circular points show the extent of the 5^th^ and 95^th^ percentiles. Points outside this range are omitted for clarity.

Rock temperatures (Figure 1) differed significantly between dry and rain forest sites at the times frogs emerged from their diurnal retreat sites, between 6–7 pm. (Mann-Whitney U test, z = −3.58, P<0.001, n = 18). Dry forest substrate temperatures were commonly 30°C or greater (mean maximum temperature = 31.8±2.46) when frogs emerged from retreat sites, however maximum rainforest substrate temperatures were never above 20°C (mean maximum temperature = 17.5°C±0.93).

**Figure 2:**
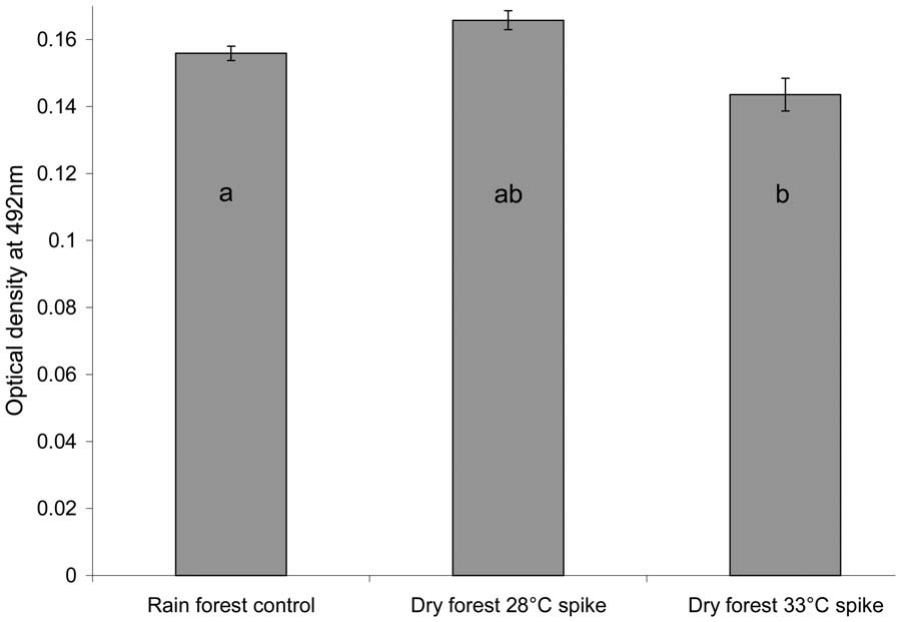
Mean adjusted optical density of *Batrachochytrium dendrobatidis* cultures in three temperature regimes. Error bars delineate standard errors of the means. Letters indicate homogeneous subsets according to Tukey's HSD post-hoc test. Rain forest control = constant 15°C; Dry forest 28°C spike = 28°C for 1 hour · day ^−1^, 15°C for 23 hours · day^−1^; Dry forest 33°C spike = 33°C for 1 hour · day^−1^, 15°C for 23 hours · day^−1^. Growth in the 33°C spike treatment was significantly lower than in both the rain forest and the 28°C spike treatments, but growth in the 28°C spike treatment did not differ significantly from that in the rain forest treatment.

After four days, cultures in all treatments had developed into dense sporangial aggregations. One-way ANOVA (F_2,87_ = 10.11, P<0.001) indicated that *Bd* growth rates differed among the three thermal treatments. Tukey's tests (Figure 2) indicated that the dry forest 33°C temperature spike cultures had significantly lower *Bd* growth (mean adjusted OD_492_) than those in either the rain forest control or the dry forest 28°C spike treatment. The growth of *Bd* did not differ significantly between rain forest control and dry forest 28°C spike treatment. Light microscopy of cultures during the trial did not reveal any observable differences in the development time of sporangia.

## Discussion

Carey et al. [19] demonstrated that in highly susceptible species, the development of chytridiomycosis can be explained as a consequence of unregulated exponential growth of the population of *Bd* on the host. After multiple generations of exponential growth, even small differences in the growth rate parameter can make very large differences in population size. The reduction of *Bd* growth we detected with short-term elevation of temperature in the dry forest 33°C spike treatment was statistically significant, but was relatively small, and visual observation of cultures under light microscopy suggested that zoospores developed into mature sporangia over similar periods of time across the three treatments. This suggests that the lower density of cultures exposed to 33°C temperature spikes was caused by lower survival or settlement rates of zoospores, or lower production of zoospores per sporangium. Although our experiment encompassed only a single *Bd* life cycle, the small difference we observed in the population growth rate could translate to large differences in population size with time later in the population growth trajectory, and might account for the lower *Bd* virulence observed by Puschendorf et al. [15] at dry forest field sites. If the temperature responses of the *Bd* that occurs at the dry forest field site are similar to those of the *Bd* isolate used in this experiment, night “basking” on warmed rocks could explain the persistence of *Litoria nannotis* and *L. lorica* with *Bd* in dry forest habitats.

There are at least two alternative explanations for the persistence of *Litoria nannotis* and *L. lorica* with *Bd* at dry forest sites. First, frogs at two dry forest sites spent more time under running water than did those at rain forest sites [16]. This may have flushed away zoospores that would otherwise re-infect frogs and boost infection intensity towards a lethal threshold. Second, warmer temperatures may stimulate amphibian immune defenses [23], [24], [25], but there are few studies on the effects of short-term warming on immune function.

The response of *Bd* to temperature *in vivo* and/or in natural environments could differ from the responses we observed *in vitro*. We did not perform *in vivo* experiments due to the conservation status of our species, but our results are in accord with those of Richards-Zawacki [21], who found that the mean of the body temperatures of uninfected wild *Atelopus zeteki* individuals was higher than that of infected individuals.

It remains unclear why the amphibian-*Bd* symbiosis is shifted at least partway along the axis from host-pathogen to host-commensal in dry forest *Litoria* populations. Short-term temperature spikes over longer time scales (several weeks) than were possible in our experiment may reduce *Bd* growth rates enough to allow host defenses to preclude the development of lethal infection intensities. We suggest that detailed data on daily body temperatures of individuals should ultimately be used to determine the conditions in which to carry out *in vitro* and *in vivo* experiments over longer periods that will illustrate the exact nature of thermal effects on the interaction between amphibians and *Bd*. Future work should also test the effects of realistic thermal regimes on amphibian immune function. Experiments could also be used to test the effects of immersion in running water. Understanding the specific factors driving context-dependency in *Litoria-Bd* symbioses would improve prediction of future chytridiomycosis-driven declines and location of refugia for threatened species, like that already found for *Litoria lorica*.
